# Mobilizing the bystanders: How social capital drives user reporting on social media harmful content

**DOI:** 10.3389/fpsyg.2026.1785107

**Published:** 2026-07-13

**Authors:** Hong Zhou, Yan Ye

**Affiliations:** 1 School of Artificial Intelligence and Big Data, Wuhan Business University, Wuhan, China; 2 Library, Northwest A&F University, Yangling, China

**Keywords:** harmful content, online bystander intervention, social capital, social media platforms, user reporting

## Abstract

User reporting is a critical governance mechanism for addressing harmful content, such as misinformation, cyberbullying, and hate speech, on social media platforms. Prior research, largely grounded in the online bystander intervention framework, has emphasized users’ situational responses to specific incidents, paying relatively less attention to the enduring social relationships that embed reporting behavior within platform communities. This study draws on social capital theory to conceptualize user reporting as a socially embedded practice. Structural social capital, reflected in social interaction ties; relational social capital, reflected in identification; and cognitive social capital, reflected in value congruence, are theorized to shape users’ sense of belonging, altruistic motivation, and reporting self-efficacy, three more immediate psychological mechanisms that help translate communal resources into reporting engagement. Data from a two-wave online survey conducted across multiple Chinese social media platforms were analyzed using partial least squares structural equation modeling (PLS-SEM). The findings show that social interaction ties, identification, and value congruence are associated with reporting engagement mainly through these psychological mechanisms. This work extends online bystander intervention research by demonstrating how users’ social capital in platform communities underpins sustained reporting participation, and it offers practical insights for designing reporting systems that leverage community-based social resources.

## 1 Introduction

The rapid proliferation of harmful content on social media platforms—including misinformation, cyberbullying, and hate speech—has increasingly undermined individual well-being and the quality of public discourse, raising urgent concerns for platform governance (Chan et al., 2021; Lazer et al., 2018). As users are routinely exposed to such content, their reactions as bystanders collectively shape whether harmful content spreads, persists, or is curtailed. In response, platforms have adopted a range of content moderation strategies, with user reporting—where individuals flag harmful content for review—emerging as a critical component of the broader content governance ecosystem (Crawford and Gillespie, 2016; Morrow et al., 2022). Scholars have highlighted its potential not only to enhance algorithmic moderation but also to curtail harmful content through decentralized participation (Gwebu et al., 2022). Notwithstanding this recognized importance, understanding what drives users to report remains an urgent question.

Much of the existing research on user reporting draws on the online bystander intervention framework (Darley and Latané, 1968). This perspective highlights how situational social cues, especially the presence of others, can inhibit intervention through mechanisms such as diffusion of responsibility and evaluation apprehension (Gimpel et al., 2021; Wong et al., 2021). Accordingly, many studies have examined how incident-level factors, such as the number of other bystanders or the visibility of moderators, shape users’ willingness to report harmful content (Bhandari et al., 2021; Wong et al., 2021). These studies have provided valuable insight into the immediate, situation-specific drivers of reporting.

However, the bystander perspective, as predominantly applied, tends to conceptualize reporting as a discrete reaction to an isolated incident, giving less attention to the ongoing social relationships and community attachments that embed reporting within the broader fabric of platform life. Yet, user reporting is rarely a one-off act; it often occurs within communities where users have developed enduring interaction patterns, relational bonds, and shared values. Consequently, a purely situational account leaves unexplained how users’ sustained social embeddedness in platform communities conditions their reporting engagement.

To address this gap, we draw on social capital theory (Nahapiet and Ghoshal, 1998), which highlights how structural, relational, and cognitive resources embedded in social networks shape individual behavior. In social media harmful content reporting, these dimensions correspond to users’ social interaction ties, community identification, and value congruence with the platform. These forms of social capital capture users’ enduring embeddedness in platform communities and provide a foundation for reporting engagement.

We further theorize that social capital influences reporting engagement through proximal psychological mechanisms. Online bystander intervention research suggests that witnessing harmful content does not automatically lead to action; instead, intervention depends on users’ concern for others (altruistic motivation), their connection to the relevant community (sense of belonging), and their perceived ability to intervene effectively (reporting self-efficacy). We therefore focus on altruism, sense of belonging, and reporting self-efficacy as three mechanisms through which social capital promotes reporting engagement. Each mechanism is theoretically linked to a specific social capital dimension: social interaction ties and identification primarily foster belonging, whereas identification and value congruence enhance altruism and self-efficacy. To test this model, we conducted a two-wave online survey across major Chinese social media platforms and analyzed the data using partial least squares structural equation modeling (PLS-SEM).

This study offers three principal contributions. First, it advances the literature on digital bystander intervention by shifting from a predominantly situational focus to a socially embedded account, demonstrating that users’ reporting engagement is systematically shaped by the social capital they accumulate within platform communities. Second, it enriches social capital theory in the context of digital platform governance by specifying the psychological pathways through which social resources translate into prosocial reporting behavior. Third, the findings highlight actionable implications for platform design, suggesting that strategies cultivating social interaction ties, identification, and value congruence can foster a sustained culture of user reporting participation in content governance.

## 2 Theoretical background

### 2.1 Bystander intervention framework and content reporting on social media platforms

The bystander intervention framework (Darley and Latané, 1968) provides the dominant lens for understanding why individuals do or do not help others in need. According to this framework, intervention depends on a five-step decision process: noticing an incident, interpreting it as requiring help, assuming personal responsibility, deciding how to help, and implementing the chosen action. At each step, the presence of others can inhibit intervention through diffusion of responsibility, pluralistic ignorance, and evaluation apprehension. Applied to social media, this perspective casts user reporting of harmful content as a situational judgment made under the influence of social cues.

Evidence for situational bystander effects on social media harmful content reporting is well-documented. Classic studies found that the mere presence of other users reduces individuals’ willingness to report harassment (Wong et al., 2021) or flag uncivil comments (Naab et al., 2018). Yet recent empirical work paints a more nuanced picture. Bhandari et al. (2021) demonstrated that visible moderators and pro-reporting social norms increase cyberbullying flagging; Record et al. (2020) reported that stronger subjective norms predict users’ intention to report self-harm-related posts; Gimpel et al. (2021) demonstrated that injunctive social norms significantly increase reporting rates for fake news; Obermaier and Schmid (2026) demonstrated that pro-social communication norms on platforms indirectly promote actual bystander intervention (including reporting) by strengthening perceived pro-social intervention social norms and personal responsibility; and Saldana and Vu (2022) found that perceptions of audience engagement encourage debunking and reporting of fake news. Similarly, Kim et al. (2022) showed that upvotes of counterspeech comments reduce uncertainty and clarify users’ intentions to report sexist hate speech. Table 1 summarizes representative works linking various peer-related social cues to reporting-related responses.

**Table 1:** Studies on peer-related social cues in reporting harmful content on social media.

Reference	Type of harmful content	Theory	Peer-related social cues	Dependent variable	Findings
Naab et al. (2018)	Uncivil user comments	Bystander intervention model (BIM)	Response disagreement	Flagging behavior	Response disagreement decreases flagging likelihood only in conditions of an impolite response.
Record et al. (2020)	Self-harm-related posts	Diffusion of innovation theory; Theory of planned behavior	Subjective norms	Intention to report	The stronger the subjective norms influenced by others, the more likely the user is to intend to report.
Wong et al. (2021)	Social media harassment	Bystander intervention model (BIM); Appraisal theories	Presence of others	Willingness to use reporting functions	The presence of others reduces users’ willingness to report.
Gimpel et al. (2021)	Fake news	Social norms theory	Injunctive social norm messages and descriptive social norm messages	Amount of reported fake news	Injunctive norm alone significantly increased odds of reporting fake news; descriptive norm alone not significant; combination was strongest; strength of descriptive norm had an inverted U-shaped effect on reporting; combined condition also increased false reporting of real news but overall net effect positive.
Bhandari et al. (2021)	Cyberbullying	Bystander intervention model (BIM)	Moderator visibility; Social norms	Flagging behavior	Both the visibility of moderators and the social norms established by other users play a crucial role in promoting user-reporting behavior.
Saldana and Vu (2022)	Misinformation	--	Perception of audience engagement	Debunk misinformation	The engagement of others on social media platforms positively influences users’ debunking behavior, including their willingness to report misinformation.
Kim et al. (2022)	Sexist hate speech	Information uncertainty; Gender bias	Upvotes of the counterspeech	Intention to report hate speech comment	The more likes a comment receives in response to hate speech, the clearer the user’s intention to report it.
Obermaier and Schmid (2026)	Online hate speech	Bystander intervention model (BIM), social norms theory	Platform’s descriptive communication norm	Actual bystander intervention behavior (indirect: reporting)	Prosocial norm did not directly affect intervention but indirectly increased it via perceived norms and personal responsibility.

These studies collectively underscore that social context on digital platforms is more than a diffuse inhibitor; it can also motivate and shape reporting. However, the emphasis remains largely on incident-specific, situational factors—visible peer reactions, perceived norms, or immediate cues—rather than the enduring social relationships and community structures that surround users over time. As a result, the bystander literature, while rich in situational dynamics, has not systematically addressed how users’ ongoing ties, attachments, and shared understandings with a platform community condition their readiness to intervene by reporting harmful content. We argue that user reporting is not merely a situational response but a socially embedded practice shaped by the social capital users accumulate within platform communities.

### 2.2 Social capital in digital platforms

Social capital theory posits that actors derive value from the resources embedded in, available through, and shaped by their network of relationships (Nahapiet and Ghoshal, 1998). These resources are commonly organized into three interrelated dimensions: structural capital (the configuration of connections and interaction patterns), relational capital (the quality of relationships, including trust, identification, and reciprocity), and cognitive capital (shared interpretations, values, and meaning systems that enable coordinated action). While this tripartite framework has been widely applied in organizational and community contexts, its systematic application to understanding bystander intervention against harmful content on digital platforms remains underdeveloped.

Prior research on online bystander intervention has examined factors that resonate with each dimension of social capital, yet these contributions remain fragmented and have not been consolidated into a coherent theoretical framework. We re-examine social capital in the specific context of harmful content bystander intervention and identify the most parsimonious constructs that best represent each dimension, Table 2 summarizes the conceptualization adopted in this study.

**Table 2:** Social capital in harmful content reporting on social media platforms.

Social capital dimension	Core construct (this study)	Definition	Sources
Structural social capital	Social interaction ties	The strength, frequency, and density of users’ connections within a platform’s interaction network.	Visibility of exchange patterns (Barlinska et al., 2013); Social ties (Bastiaensens et al., 2014); Relative social standing of involved parties (Ireland et al., 2020); Presence of others (Wong et al., 2021); Moderator visibility (Bhandari et al., 2021); Type of victim–bystander relationship (Chen, 2024); Intergroup contact (Abbott et al., 2026).
Relational social capital	Identification	The emotional attachment and sense of oneness users experience toward the platform community.	Collective efficacy (Ziegele et al., 2020); Provision of online social support (Stehr, 2023); Quality of social relationships with uncivil users (Gagrcin, 2024); Social bonds and attachment (Chen et al., 2025); Ideological similarity (Lu and Luqiu, 2025).
Cognitive social capital	Value congruence	The degree to which a user perceives alignment between their personal values and the values, norms, and governance principles espoused by the platform community.	Visibility of community rules (Matias, 2019); Shared understanding of rules and structures (Ziegele et al., 2020); Injunctive and descriptive social norm (Gimpel et al., 2021); Social norms (Bhandari et al., 2021); Subjective norms (Achuthan et al., 2022); Platform communication norms that define acceptable behavior (Obermaier and Schmid, 2026); Collective and injunctive norms (Alcántara-Lázaro et al., 2026).

Studies have examined how users’ network embeddedness shapes their intervention intentions, focusing on factors such as the nature of social ties (Bastiaensens et al., 2014), the type of victim–bystander relationship (Chen, 2024), intergroup contact (Abbott et al., 2026), the visibility of exchange patterns (Barlinska et al., 2013), peer presence (Wong et al., 2021), moderator visibility (Bhandari et al., 2021), and the relative social standing of the parties involved (Ireland et al., 2020). Collectively, these factors capture the structural dimension of social capital, which concerns the overall configuration of social connections that enables resource flows. We synthesize this set of structural indicators into the construct of *social interaction ties, defined as the strength, frequency, and density of users’ connections within a platform’s interaction network* (Wang et al., 2016). Such ties embed users in visible patterns of social exchange, thereby heightening accountability and making collective responses to harmful content more observable. Prior research suggests that platforms characterized by more frequent and diverse interactions foster stronger network embeddedness, which, in turn, shapes prosocial outcomes (Chen, 2024; Machackova, 2020).

Similarly, the relational dimension of social capital has been invoked implicitly through constructs such as social bonds and attachment (Chen et al., 2025), ideological similarity (Lu and Luqiu, 2025), quality of social relationships with uncivil users (Gagrcin, 2024), collective efficacy reflecting relational trust (Ziegele et al., 2020), and the provision of online social support (Stehr, 2023). Although these constructs are not identical, they all point to relational resources that connect users to other members and to the broader community. Rather than treating these as isolated predictors, we view them as relational conditions through which users may develop affective and self-definitional bonds with the platform community. We therefore adopt *identification, the emotional attachment and sense of oneness users experience toward the platform community* (Chang and Chuang, 2011), as the representative construct for relational social capital. Prior research suggests that such relational attachment increases users’ likelihood of intervening against cyberbullying and other harmful content (Bastiaensens et al., 2014; Gagrcin, 2024).

The cognitive dimension of social capital is evident in bystander research that highlights the role of shared normative and interpretive frameworks. Existing studies have considered subjective norms as perceived social expectations (Achuthan et al., 2022), shared understandings of rules and platform structures that support coordinated action (Ziegele et al., 2020), injunctive and descriptive norms that promote reporting (Bhandari et al., 2021; Gimpel et al., 2021), platform communication norms that delineate acceptable conduct (Obermaier and Schmid, 2026), the visibility of community rules (Matias, 2019), and collective and injunctive norms that shape bystander responses (Alcántara-Lázaro et al., 2026). Despite their conceptual differences, these factors collectively capture users’ shared understandings of what is appropriate, expected, and collectively valued within a platform community. We integrate these related insights through the construct of *value congruence, referring to the perceived alignment between users’ personal values and the values, norms, and governance principles espoused by the platform* (Tsai and Pai, 2021). Higher value congruence should facilitate the internalization of platform norms, making users more likely to view reporting harmful content as a legitimate and socially responsible form of intervention. This argument is consistent with prior work showing that shared values and rule understandings strengthen bystanders’ perceived obligation to intervene in harmful online interactions (Gagrcin, 2024; Machackova, 2020).

By systematically consolidating these scattered fragments into the representative constructs of social interaction ties, identification, and value congruence, we provide a parsimonious yet theoretically grounded account of the social resources that underpin users’ engagement in digital platforms.

## 3 Research model and hypotheses development

### 3.1 Research model

Building on social capital theory, we do not treat it as a substitute for the online bystander intervention perspective. Rather, we use social capital theory to explain why some users are more likely than others to develop the psychological conditions that support reporting engagement when they encounter harmful content on digital platforms. The bystander intervention model suggests that people do not automatically move from witnessing a problematic incident to taking action. Instead, intervention unfolds through a process in which individuals notice the incident, interpret it as requiring intervention, assume responsibility, know how to help, and implement the helping action. In platform reporting, the first two steps concern whether users recognize harmful content as problematic and warranting attention. Because action implementation corresponds to the outcome of interest, we focus on the psychological conditions implied by the intermediate steps before action implementation.

We organize these conditions into three proximal mechanisms. The first two elaborate the responsibility-assumption step, which online bystander research has shown to be central to intervention. In platform communities, assuming responsibility for reporting may stem from users’ social embeddedness in the community and from their other-oriented concern for those affected by harmful content. Sense of belonging captures the former mechanism: users who feel connected to the platform community are more likely to view threats to the community as personally relevant and to experience a responsibility to help maintain it (Nowell and Boyd, 2010). Altruism captures the latter mechanism: users who are concerned about others’ welfare are more likely to feel motivated to protect those who may be harmed by harmful content (Stern et al., 1999). The third mechanism reflects the capability-related step of knowing how to help. Reporting self-efficacy captures users’ belief that they know how to report harmful content and can do so effectively.

*Altruism* captures users’ willingness to protect others and the broader online community from harmful content (Mahmood et al., 2019). Because reporting often requires time and effort and may provide no direct personal benefit (Gimpel et al., 2021), altruistic users are more likely to engage in reporting to protect victims, vulnerable users, and the collective good of the community.

*Sense of belonging* reflects the extent to which users feel accepted, connected, and psychologically situated within the platform community (Zhao et al., 2012). Because reporting harmful content helps maintain the social environment of a community, users with a stronger sense of belonging are more likely to view harmful content as a threat to a valued collective space and to see reporting as a contribution to the well-being and order of that community.

*Reporting self-efficacy* refers to users’ belief that they can successfully identify harmful content, use the reporting tools provided by the platform, and make a meaningful contribution through reporting (Tsai and Pai, 2021; Zhou et al., 2024). Users may remain passive even when they care about others and feel connected to the community if they do not know how to report, doubt whether reporting is worth the effort, or question whether their action will have any effect. Reporting self-efficacy therefore captures a key enabling condition for intervention in platform environments governed by specific interfaces, rules, and procedures.

**Figure 1:**
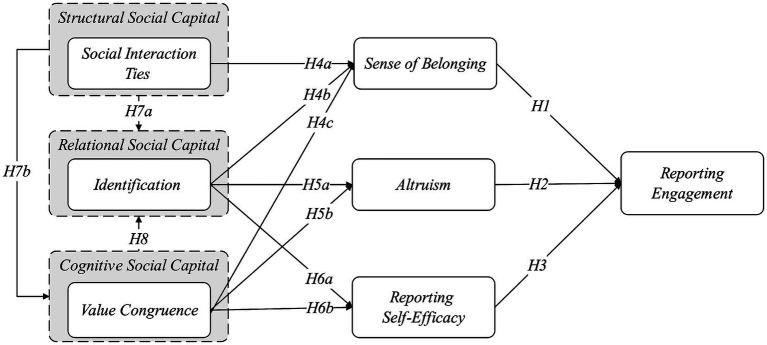
Research model.

Taken together, these three mechanisms explain how social capital may be translated into reporting engagement. Social interaction ties expose users to ongoing exchanges, repeated contact, and greater awareness of other users and community conditions. Identification reflects users’ relational attachment to the platform community and can strengthen their willingness to care about and act for that community. Value congruence reflects alignment between users’ own values and the norms, rules, and governance principles of the platform community, thereby reinforcing the perceived appropriateness and meaningfulness of reporting harmful content. Accordingly, social interaction ties, identification, and value congruence are positioned as social capital antecedents that shape altruism, sense of belonging, and reporting self-efficacy, which in turn predict reporting engagement. Figure 1 presents the research model, and the specific hypothesized relationships are developed in the next section.

### 3.2 Psychological mechanisms and reporting engagement

When users experience a strong sense of belonging to a platform community, they are more likely to perceive harmful content as relevant to a shared social environment in which they are personally situated. Rather than viewing such content as a problem concerning only distant others, they may see it as undermining the quality, safety, and normative order of a community space to which they feel connected. This perceived relevance can increase their sense of personal responsibility to help address the problem.

This reasoning is consistent with the bystander intervention perspective, which suggests that individuals are more likely to intervene when they feel personally responsible for responding to a problematic situation and are less likely to assume that others will act. In the context of platform reporting, reporting offers users a practical way to fulfill this responsibility by alerting the platform to harmful content and activating its governance mechanisms. Thus, users with a stronger sense of belonging are more likely to transform their awareness of harmful content into sustained reporting engagement, because they experience the platform community as a shared space whose well-being partly depends on members’ actions. We therefore hypothesize:

*H1*: A sense of belonging is positively associated with reporting engagement.

Users who are more altruistically oriented are more likely to view harmful content as something that should be addressed for the sake of others and the broader platform community. In digital bystander contexts, this other-oriented motivation encourages users to move beyond passive awareness and toward action, even when reporting requires time or effort (Gimpel et al., 2021). Consistent with prior research showing that empathic concern and other-oriented values promote helping and prosocial intervention behaviors, altruistic users are more likely to perceive reporting as a meaningful way to protect victims and contribute to a safer online environment. In this sense, altruism functions as an important motivational driver that turns concern for others into concrete reporting engagement. We therefore hypothesize:

*H2*: Altruism has a positive impact on reporting engagement.

The bystander intervention perspective suggests that intervention depends not only on prosocial motivation but also on the confidence that one knows how to respond and can do so effectively. In the context of harmful-content reporting, users with higher reporting self-efficacy should be less constrained by uncertainty about platform rules, hesitation about how to use reporting tools, or doubt about whether their actions will make any difference (Zhou et al., 2024). As a result, they are more likely to translate their recognition of harmful content and their intention to respond into actual reporting behavior. This may be particularly important in digital environments, where reporting procedures differ in complexity and transparency across platforms. We therefore hypothesize:

*H3*: Reporting self-efficacy has a positive impact on reporting engagement.

### 3.3 Social capital as antecedents of psychological mechanisms

The three dimensions of social capital serve as social resources that cultivate sense of belonging. Social interaction ties embed users in ongoing streams of communication and exchange. Frequent and intensive interaction increases users’ embeddedness in the platform network, reinforcing their perception of being part of an enduring social system (Kim, 2018). This heightened embeddedness fosters a sense of belonging. Likewise, identification captures emotional attachment to the community and strengthens users’ commitment to the group (Bhandari et al., 2021). Value congruence reduces interpretive distance and enhances psychological coherence (Kim, 2018; Zhao et al., 2012). Together, these dimensions contribute to users’ sense of belonging. Thus, all three dimensions are theorized to serve as antecedents of sense of belonging:

*H4a*: Social interaction ties are positively associated with users’ sense of belonging.

*H4b*: Identification is positively associated with users’ sense of belonging.

*H4c*: Value congruence between users and the platform is positively associated with users’ sense of belonging.

Altruism in the reporting context reflects an other-oriented motivation to protect others from harm. Such motivation may be grounded not only in empathic concern, an affective response to others’ distress (Batson and Shaw, 1991), but also in internalized moral standards regarding the welfare of the community. Two social capital dimensions are particularly relevant for cultivating this altruistic motivation.

Identification may foster altruism by reducing psychological distance between users and other members of the platform community (Batson, 1991). When users identify with a platform community, other members are less likely to be perceived as anonymous strangers and more likely to be seen as socially connected community members whose welfare deserves consideration. Such perceived connectedness can make the vulnerability of potential victims more salient and facilitate perspective-taking and empathic concern toward those who may be harmed by harmful content. In this way, identification broadens users’ circle of moral concern within the community and strengthens their willingness to act for the benefit of others. Accordingly, users with stronger identification are more likely to develop altruistic motivation to report harmful content.

Value congruence amplifies altruism by imbuing prosocial action with moral significance. When users perceive that the platform’s values align with their own, they are more likely to view protecting the community from harm as a personally meaningful endeavor. This reasoning is consistent with prior work suggesting that psychological meaningfulness elicited by value congruence can serve as an internal guideline that engages individuals more completely and motivates them to invest greater time and effort in social group interactions (Tsai and Pai, 2021). In the reporting context, such alignment activates internalized standards of conduct and a sense of moral obligation, thereby driving altruistic tendencies even in the absence of an immediate relational tie to those affected by harmful content. Therefore:

*H5a*: Identification positively influences altruism.

*H5b*: Value congruence positively influences altruism.

Identification may enhance users’ reporting self-efficacy by strengthening their perceived embeddedness and sense of agency within the platform community. Self-efficacy refers to individuals’ belief in their capability to organize and execute the actions required to achieve desired outcomes. In the context of reporting harmful content, users may hesitate not only because they are uncertain about what to report, but also because they doubt whether their action will be noticed, supported, or consequential. When users identify with a platform community, they are more likely to perceive themselves as legitimate community members whose actions matter to the functioning of the community. This sense of membership can provide efficacy-relevant cues, such as perceived social support, anticipated validation from other members, and confidence that the platform community will take such reports seriously. These cues reduce perceived futility and strengthen users’ belief that they can perform reporting in a way that contributes to effective moderation. Accordingly, users with stronger identification are more likely to develop higher reporting self-efficacy.

Value congruence supports efficacy by creating a perception of normative support. Users who see their personal values reflected in the platform are more likely to experience meaningfulness and self-congruence when engaging in reporting (Tsai and Pai, 2021). This alignment reduces role ambiguity and enhances the conviction that their reporting efforts are warranted and likely to be effective. Together, these relational and cognitive antecedents nurture a robust sense of reporting self-efficacy. We therefore hypothesize:

*H6a*: Identification positively influences users’ reporting self-efficacy.

*H6b*: Value congruence positively influences users’ reporting self-efficacy.

### 3.4 Relationships among social capital dimensions

Extensive research has shown that the structural, relational, and cognitive dimensions of social capital are interdependent. For example, structural capital positively influences relational capital and cognitive capital (Wang et al., 2016; Zhao et al., 2012). In the context of harmful content reporting on social media, social interaction ties may foster both identification and value congruence. Repeated interactions provide users with opportunities to communicate with others, observe community responses, and participate in shared practices. These interactions can generate emotional attachment and a sense of psychological connection to the platform community, thereby strengthening user identification. At the same time, frequent interactions expose users to community norms, expectations, and evaluative standards, which can enhance their perception that their own values are aligned with those of the platform community.

Value congruence may also strengthen user identification. When users perceive that their values are consistent with those of the platform community, they are more likely to view the community as self-relevant and to incorporate it into their social identity. Shared values therefore provide a cognitive foundation for emotional attachment and a sense of oneness with the community. Thus:

*H7a*: Social interaction ties are positively associated with user identification.

*H7b*: Social interaction ties are positively associated with value congruence.

*H8*: Value congruence is positively associated with user identification.

## 4 Research method

### 4.1 Research design and setting

The study was conducted in the context of widely used Chinese social media platforms, including Sina Weibo, Zhihu, Xiaohongshu, TikTok, and Bilibili. These platforms maintain comparable content-reporting policies and operational features, offering a consistent environment in which users encounter harmful content and can choose to report it. To ensure ecological validity, the survey explicitly targeted users who had previously reported harmful content on at least one of these platforms. A two-wave data collection design was employed to mitigate common method bias and temporal ambiguity: all exogenous and mediating constructs—social interaction ties (SIT), identification (IDF), value congruence (VC), sense of belonging (SOB), altruism (AT), and reporting self-efficacy (RSE)—were measured in the first wave, while the dependent variable, reporting engagement (RE), was collected 2 weeks later in a second survey.

### 4.2 Procedure and data collection

Data were collected between November 2 and November 26, 2021, a period during which platform-based reporting systems were well established. Questionnaires were randomly disseminated across the five platforms, and only respondents who confirmed prior reporting experience were eligible to participate. All participants were provided with an informed consent statement outlining the study’s purpose, voluntary participation, anonymity, and use of data solely for academic research. The questionnaire comprised three sections: an orientation to the reporting context, demographic and screening items, and the focal construct measures. To verify eligibility, respondents specified the platforms on which they had reported and described their reasons for reporting; inconsistent or dishonest responses were excluded. Data quality was further ensured by removing responses with completion times beyond three standard deviations from the mean (Meade and Craig, 2012). After screening steps, 339 valid responses were retained for analysis. An *a priori* power analysis using G*Power 3.1 (Faul et al., 2007), assuming a medium effect size (*f*^2^ = 0.15), a significance level of 0.05, and a statistical power of 0.80 for a model with six predictors, indicated a minimum required sample size of approximately 98; thus the final sample of 339 provided adequate statistical power.

### 4.3 Measures

All constructs were measured using multi-item scales adapted from previously validated instruments. Each item was rated on a seven-point Likert scale anchored at 1 (“strongly disagree”) to 7 (“strongly agree”). The full set of items appears in the Appendix.

Reporting engagement was assessed using items adapted from Schaufeli et al. (2017) and Tsai and Pai (2021). The scale captures users’ affective and state-based involvement in reporting activities, including feelings of vitality and focused involvement when reporting harmful content. Social interaction ties were measured based on the scale developed by Wang et al. (2016). The items reflect the frequency, continuity, and repetitiveness of users’ interactions on the platform, indicating the strength of ongoing interpersonal connections. Identification was assessed with items adapted from Chang and Chuang (2011). This construct reflects users’ perceived emotional attachment and sense of unity with the virtual community, including feelings of connectedness, stable relationships, and positive attitudes toward the platform community. Value congruence was derived from Tsai and Pai (2021). The items evaluate the perceived alignment between users’ personal values and the values embedded in the platform’s rules and reporting mechanisms, particularly with regard to the principles and relevance of reporting harmful content. Altruism was adapted from Mahmood et al. (2019). It captures users’ concern for the well-being of others and their intrinsic willingness to consider how content affects other users and to engage in reporting for collective benefit. Sense of belonging was assessed using items from Zhao et al. (2012). The items measure users’ feelings of membership and involvement in the platform, capturing the degree to which they perceive themselves as part of the platform community. Reporting self-efficacy was measured with scales from Tsai and Pai (2021). This construct reflects users’ confidence in their ability to report harmful content effectively.

A pretest with four information systems experts evaluated the face and content validity of the questionnaire; minor adjustments were made to clarify item wording and alignment with constructs.

### 4.4 Common method bias assessment

Two procedures were used to assess potential common method variance. First, Harman’s single-factor test using principal component analysis (Podsakoff et al., 2003) showed that the largest factor explained 33.01% of the total variance, well below the 50% threshold, suggesting no single factor dominated the data. Second, a marker variable technique (Lindell and Whitney, 2001) employed learning cost (see Appendix) as a theoretically unrelated marker; all correlations between the marker variable and the dependent variable were below 0.3. These results indicate that common method bias is unlikely to pose a serious concern. Additionally, all variance inflation factor (VIF) values for both indicators and constructs were below the recommended threshold of 3.3 (Diamantopoulos and Siguaw, 2006), confirming the absence of significant multicollinearity.

## 5 Data analysis and results

The research model was tested using partial least squares structural equation modeling (PLS-SEM), a variance-based technique suited for complex models and exploratory contexts (Hair et al., 2019). Bootstrapping with 5,000 resamples was used to evaluate the significance of all path coefficients.

### 5.1 Measurement model

A confirmatory factor analysis (CFA) was conducted to assess reliability and validity following established guidelines (Hair et al., 2019). Results are summarized in Table 3.

**Table 3:** Measurement reliability and validity.

Construct	Item	Loading	CR	AVE	SIT	IDF	VC	SOB	AT	RSE	RE
SIT	SIT1	0.813	0.878	0.642	**0.801**						
	SIT2	0.779									
	SIT3	0.820									
	SIT4	0.793									
IDF	IDF1	0.786	0.875	0.638	0.645	**0.798**					
	IDF2	0.822									
	IDF3	0.813									
	IDF4	0.772									
VC	VC1	0.829	0.823	0.608	0.321	0.350	**0.780**				
	VC2	0.717									
	VC3	0.790									
SOB	SOB1	0.863	0.865	0.762	0.450	0.521	0.276	**0.873**			
	SOB2	0.882									
AT	AT1	0.774	0.748	0.597	0.297	0.404	0.263	0.540	**0.773**		
	AT2	0.771									
RSE	RSE1	0.816	0.774	0.632	0.167	0.258	0.409	0.215	0.252	**0.795**	
	RSE2	0.773									
RE	RE1	0.835	0.867	0.684	0.295	0.380	0.289	0.512	0.495	0.364	**0.827**
	RE2	0.818									
	RE3	0.829									

Bold-faced diagonal elements are the square roots of AVEs. SIT = Social Interaction Ties; IDF = Identification; VC = Value Congruence; SOB = Sense of Belonging; AT = Altruism; RSE = Reporting Self-Efficacy; RE = Reporting Engagement.

Reliability was evaluated using composite reliability (CR) and indicator loadings. All CR values exceeded 0.7, and each factor loading was above 0.708, demonstrating internal consistency. Convergent validity was established, as all average variance extracted (AVE) values surpassed the 0.5 threshold. Discriminant validity was assessed with the Fornell-Larcker criterion (Fornell and Larcker, 1981): the square root of each construct’s AVE (diagonal elements in Table 3) exceeded its correlations with all other constructs, confirming adequate discriminant validity.

### 5.2 Structural model

**Figure 2:**
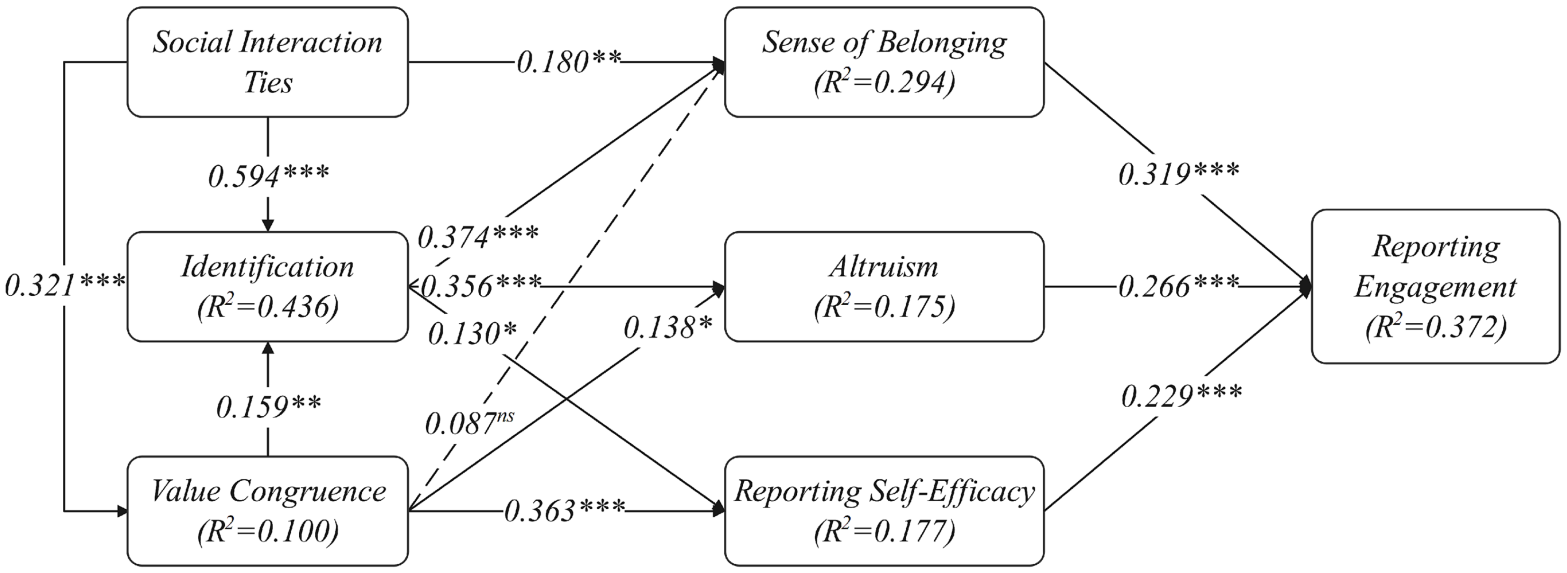
Structural model results.

Model fit was evaluated using the standardized root mean square residual (SRMR), which was below the 0.08 threshold, indicating acceptable fit (Henseler et al., 2016). Path coefficients, t-statistics, and confidence intervals were obtained through bootstrapping and are displayed in Table 4 and Figure 2.

**Table 4:** Hypothesis testing results.

**Hypothesis**	**Path**	***β***	***T*-statistic**	**Confidence interval**	**Supported?**
H1	SOB ➔ RE	0.319***	5.359	[0.200,0.431]	Yes
H2	AT ➔ RE	0.266***	4.825	[0.158,0.373]	Yes
H3	RSE ➔ RE	0.229***	4.676	[0.132,0.324]	Yes
H4a	SIT ➔ SOB	0.180**	2.608	[0.046,0.316]	Yes
H4b	IDF ➔ SOB	0.374***	5.134	[0.230,0.514]	Yes
H4c	VC ➔ SOB	0.087	1.240	[−0.047,0.228]	No
H5a	IDF ➔ AT	0.356***	6.372	[0.246,0.469]	Yes
H5b	VC ➔ AT	0.138*	2.032	[0.006,0.271]	Yes
H6a	IDF ➔ RSE	0.130*	2.272	[0.019,0.244]	Yes
H6b	VC ➔ RSE	0.363***	6.364	[0.248,0.470]	Yes
H7a	SIT ➔ IDF	0.594***	13.510	[0.503,0.676]	Yes
H7b	SIT ➔ VC	0.321***	6.183	[0.218,0.423]	Yes
H8	VC ➔ IDF	0.159***	3.089	[0.057,0.260]	Yes

*p < 0.05, **p < 0.01, ***p < 0.001. SIT = Social Interaction Ties; IDF = Identification; VC = Value Congruence; SOB = Sense of Belonging; AT = Altruism; RSE = Reporting Self-Efficacy; RE = Reporting Engagement.

All hypothesized relationships were supported except H4c. Sense of belonging (*β* = 0.319, *t* = 5.359, 95% CI [0.200, 0.431]), altruism (*β* = 0.266, *t* = 4.825, 95% CI [0.158, 0.373]), and reporting self-efficacy (*β* = 0.229, *t* = 4.676, 95% CI [0.132, 0.324]) each positively and significantly predicted reporting engagement, confirming H1, H2, and H3, respectively. The social capital dimensions exerted significant effects on the proximal psychological mechanisms: social interaction ties (*β* = 0.180, *t* = 2.608) and identification (*β* = 0.374, *t* = 5.134) were positively associated with sense of belonging, while the path from value congruence (*β* = 0.087, *t* = 1.240) was not statistically significant; thus H4a and H4b were supported but H4c was not. Identification positively affected altruism (*β* = 0.356, *t* = 6.372) and reporting self-efficacy (*β* = 0.130, *t* = 2.272), and value congruence positively influenced both altruism (*β* = 0.138, *t* = 2.032) and reporting self-efficacy (*β* = 0.363, *t* = 6.364), supporting H5a, H5b, H6a, and H6b. Within the social capital architecture, social interaction ties were positively related to identification (*β* = 0.594, *t* = 13.510) and value congruence (*β* = 0.306, *t* = 4.734), and value congruence further strengthened identification (*β* = 0.141, *t* = 2.862), thereby supporting H7a, H7b, and H8.

The explanatory power of the model was assessed through the coefficient of determination (*R*^2^). As shown in Figure 2, social interaction ties and value congruence jointly explained 43.6% of the variance in identification. Social interaction ties, identification, and value congruence together accounted for 29.4% of the variance in sense of belonging. Identification and value congruence explained 17.5% of the variance in altruism and 17.7% in reporting self-efficacy. Sense of belonging, altruism, and reporting self-efficacy collectively accounted for 37.2% of the variance in reporting engagement.

### 5.3 *Post hoc* mediation analysis

To examine the mediating roles of sense of belonging, altruism, and reporting self-efficacy, a bias-corrected nonparametric percentile bootstrapping procedure with 5,000 resamples was conducted. Results are presented in Table 5.

**Table 5:** Results of the mediation analysis.

IV	Mediator(s)	Indirect effect (bootstrap)			Direct effect			Mediation type
		Effect	LLCI	ULCI	Effect	LLCI	ULCI	
SIT	IDF ➔ AT	0.053	0.029	0.088	0.009	−0.105	0.127	Full
SIT	IDF ➔ RSE	0.016	0.003	0.035	0.009	−0.105	0.127	Full
SIT	IDF ➔ SOB	0.067	0.036	0.112	0.009	−0.105	0.127	Full
SIT	SOB	0.056	0.014	0.124	0.009	−0.105	0.127	Full
SIT	VC ➔ AT	0.011	0.001	0.029	0.009	−0.105	0.127	Full
SIT	VC ➔ IDF ➔ AT	0.005	0.002	0.010	0.009	−0.105	0.127	Full
SIT	VC ➔ IDF ➔ RSE	0.001	0.000	0.004	0.009	−0.105	0.127	None
SIT	VC ➔ IDF ➔ SOB	0.006	0.002	0.013	0.009	−0.105	0.127	Full
SIT	VC ➔ RSE	0.024	0.011	0.043	0.009	−0.105	0.127	Full
IDF	AT	0.090	0.050	0.143	0.057	−0.101	0.220	Full
IDF	RSE	0.027	0.006	0.059	0.057	−0.101	0.220	Full
IDF	SOB	0.113	0.060	0.187	0.057	−0.101	0.220	Full
VC	AT	0.035	0.004	0.080	0.037	−0.100	0.192	Full
VC	IDF ➔ AT	0.014	0.005	0.029	0.037	−0.100	0.192	Full
VC	IDF ➔ RSE	0.004	0.001	0.013	0.037	−0.100	0.192	Full
VC	IDF ➔ SOB	0.018	0.006	0.039	0.037	−0.100	0.192	Full
VC	RSE	0.075	0.035	0.129	0.037	−0.100	0.192	Full

Level of confidence = 95%, LLCI/ULCI = lower/upper limit of confidence interval. SIT = Social Interaction Ties; IDF = Identification; VC = Value Congruence; SOB = Sense of Belonging; AT = Altruism; RSE = Reporting Self-Efficacy; RE = Reporting Engagement. Dependent variable: RE.

Following the mediation criteria of Hu et al. (2021), an indirect effect was considered significant when its 95% bias-corrected confidence interval excluded zero. The mediation analysis revealed that sense of belonging, altruism, and reporting self-efficacy generally transmitted the influences of the social capital dimensions onto reporting engagement. As shown in Table 5, most indirect effects of social interaction ties, identification, and value congruence on reporting engagement through these psychological mechanisms were statistically significant, whereas their direct effects, with the mediators included, were not significant. These results provide broad support for full mediation across most examined paths. However, one sequential indirect pathway, from social interaction ties to reporting engagement through value congruence, identification, and reporting self-efficacy, was not statistically significant. Thus, the evidence does not support mediation for this specific path.

Specifically, social interaction ties exerted their influence through several fully indirect pathways, including pathways through sense of belonging, through identification to altruism, through identification to reporting self-efficacy, through identification to sense of belonging, through value congruence to altruism, through value congruence and identification to altruism, through value congruence and identification to sense of belonging, and through value congruence to reporting self-efficacy. However, the pathway from social interaction ties through value congruence, identification, and reporting self-efficacy was not supported. Identification showed full mediation through altruism, reporting self-efficacy, and sense of belonging. Value congruence also operated through altruism, reporting self-efficacy, and the downstream mediator identification. Overall, the results indicate that sense of belonging, altruism, and reporting self-efficacy largely mediate the relationships between the three dimensions of social capital and users’ reporting engagement, although the specific sequential path from social interaction ties through value congruence, identification, and reporting self-efficacy is not supported.

## 6 Discussion and implications

### 6.1 Discussion

This study set out to examine how social capital accumulated within platform communities translates into users’ engagement in reporting harmful content, a form of digital bystander intervention. The results generally support the proposed pathways. Sense of belonging, altruism, and reporting self-efficacy were each positively associated with reporting engagement, supporting that these immediate psychological mechanisms translate social resources into reporting engagement. Among the social capital dimensions, identification emerged as a particularly robust antecedent: it significantly predicted sense of belonging, altruism, and, to a lesser extent, reporting self-efficacy. Value congruence enhanced altruism and, more strongly, reporting self-efficacy, but its relationship with sense of belonging was not significant. Social interaction ties operated primarily through indirect routes, strongly predicting both identification and value congruence, and value congruence further reinforced identification. Mediation analyses provided broad support for the mediating role of the three proximal mechanisms, as most indirect effects were significant and the corresponding direct effects were not. Thus, the findings suggest that structural, relational, and cognitive resources generally become consequential for reporting when they are internalized as felt belonging, prosocial motivation, and perceived capability.

These findings advance an understanding of reporting engagement as a function of users’ enduring social embeddedness rather than a momentary reaction to isolated harmful content. While foundational bystander research has emphasized situational appraisals—such as the presence of others, perceived incident severity, or visible social signals like upvotes and moderator presence—as determinants of intervention (Bhandari et al., 2021; Naab et al., 2018; Obermaier et al., 2023; Schmid et al., 2024; Wong et al., 2021), the present results reveal a more cumulative and internalized process. This resonates with calls to consider enduring social factors in cyberbystander research (Machackova, 2020). Social interaction ties, identification, and value congruence represent enduring forms of social capital that gradually shape a user’s psychological orientation toward reporting. In particular, the supported indirect pathways suggest that repeated interactions foster relational identification and shared values, which in turn cultivate belonging, altruistic concern, and confidence in reporting, ultimately sustaining engagement.

Identification stands out as a central conduit through which social capital operates. Its significant links to belonging, altruism, and reporting self-efficacy indicate that when users define themselves as part of the platform community, they are more likely to feel protective, to care about others’ welfare, and to believe they can navigate reporting procedures. This is consistent with research linking social identity to heightened intervention intentions in online hate speech contexts (Obermaier et al., 2023). Value congruence exhibits a complementary profile: rather than generating emotional attachment, it appears to function as a cognitive and normative resource. Alignment with platform values clarifies what constitutes harmful content and strengthens users’ moral conviction to act, thereby amplifying altruistic motivation and reporting self-efficacy.

### 6.2 Theoretical implications

This study contributes to online bystander intervention research and social capital theory by offering a socially embedded account of reporting engagement. The first contribution is to shift the bystander intervention literature from a predominantly situational lens toward a socially embedded perspective. Traditional bystander research, including its digital extensions, explains intervention largely through incident-specific appraisals—such as noticing the event, interpreting it as requiring help, and taking personal responsibility (Darley and Latané, 1968). While this paradigm has proven valuable, it underexamines the enduring social contexts that make individuals feel responsible and capable in the first place. Our study demonstrates that reporting engagement is not merely a reactive judgment to a single harmful post; it is systematically cultivated by the social capital users accumulate through ongoing interactions, relational attachments, and cognitive alignment with platform communities. By showing that social capital operates as a distal antecedent whose effects are largely channeled through three proximal psychological mechanisms, we provide a complementary perspective on digital bystander intervention as a product of not only momentary situational cues but also sustained community embeddedness. This insight enriches bystander theory by situating intervention readiness within the relational infrastructure users build over time, connecting the micro-processes of reporting to the macro-structure of platform social life.

The second contribution lies in advancing social capital theory’s applicability to the digital reporting context. While the Nahapiet and Ghoshal (1998) framework has been widely used to explain knowledge sharing, collaboration, and prosocial action in online communities, its explanatory power for reporting harmful content has remained largely untested. Our study offers a systematic empirical validation of a social-psychological model in which structural, relational, and cognitive capital primarily influence reporting through proximal psychological mechanisms rather than through direct effects. This finding clarifies that social capital’s influence on reporting engagement is largely indirect and operates through mediation, although the mediation evidence is not uniform across all sequential pathways. Specifically, interaction ties, identification, and value congruence matter for reporting engagement mainly when they strengthen users’ sense of belonging, foster altruistic concern for others, and enhance confidence in their ability to report effectively. Moreover, we document the interdependence among capital dimensions themselves: structural ties reinforce both identification and value congruence, and cognitive alignment further solidifies relational bonds. This offers a nuanced view of how platform-based social capital is built and sustained. By demonstrating these pathways, we extend social capital theory into the domain of harmful content reporting.

### 6.3 Practical implications

Our findings offer practical implications for platform owners, community managers, and content governance teams. The central implication is that reporting engagement should not be managed merely as an incident-specific reaction to harmful content, but as a socially embedded form of user participation that develops through users’ accumulated experiences within platform communities.

First, platforms should cultivate sustained community embeddedness as a foundation for reporting engagement. Social interaction ties were positively associated with identification, value congruence, and sense of belonging, suggesting that repeated interaction provides an upstream relational infrastructure for user reporting. Platforms should therefore treat social interaction not only as a general engagement metric, but also as a reporting-relevant resource. Community spaces, topic-based groups, collaborative discussions, and participatory activities can help users build stable ties with others and become more attentive to the collective well-being of the platform. Such design approaches align with evidence that interaction opportunities can shape bystander behavior (DiFranzo et al., 2018). In such environments, reporting is less likely to be perceived as an isolated response to a single harmful post and more likely to be understood as part of ongoing community participation.

Second, platforms should strengthen community identification and clarify shared reporting values. Identification was associated with all three proximal psychological mechanisms. This pattern suggests that identification operates as a self-definitional resource: users who perceive the platform community as psychologically connected to the self are more likely to develop the motivational, affiliative, and efficacy-related conditions that support reporting. Platforms can support this process by framing reporting as a form of collective stewardship, showing users how their participation helps protect the community, and recognizing responsible contributions in ways that affirm users’ role as co-guardians of the platform community. At the same time, value congruence strengthened altruism and reporting self-efficacy, although it did not significantly predict sense of belonging. This suggests that shared values may function less as a direct source of emotional attachment and more as a normative and cognitive basis for action. Therefore, platforms should communicate community standards clearly and consistently, explaining what types of content violate shared norms, why reporting matters, and how reporting protects others. Research on norm communication and moral orientation supports the importance of aligning platform values with user expectations (Gagrcin, 2024; Wilhelm et al., 2020).

Third, platforms should manage structural, relational, and cognitive forms of social capital as an integrated governance system. Our findings show that these dimensions are interdependent: social interaction ties reinforce identification and value congruence, while value congruence further strengthens identification. Thus, platforms should avoid isolated interventions that target only one dimension of social capital (e.g., only interaction, identification, or value congruence). Instead, they should connect interaction opportunities, identity-building practices, and normative communication. For example, community discussions about platform norms can simultaneously promote user interaction, clarify shared values, and strengthen identification with the community. Such integrated strategies can help transform reporting from a discretionary individual act into a sustainable form of community-based governance.

## 7 Limitations and future research

This study is not without limitations. First, our empirical design relied on survey-based measures. This approach is appropriate for examining subjective factors, and we adopted procedural remedies, including temporal separation of predictor and outcome measurement and a marker variable, to reduce common method concerns. Future research could complement this design with behavioral trace data, longitudinal observations, or field experiments. Combining platform log data on actual reporting behavior with survey measures of social capital would help assess whether the social-psychological pathways identified here predict observed reporting actions over time, while experimental interventions targeting social interaction ties, identification, or value congruence could provide stronger causal evidence.

Second, our sample was drawn from users of Chinese social media platforms, a theoretically meaningful context for studying reporting under an active platform governance environment. Because platform governance models, moderation norms, and user expectations vary across institutional and cultural settings, future research could examine whether the proposed pathways generalize to other platform ecologies, such as interest-based communities, broad social networking sites, anonymous forums, or platforms that rely more heavily on algorithmic moderation. Cross-cultural comparative studies would be useful for determining whether the effects of social capital through belonging, altruism, and reporting self-efficacy remain stable across contexts or vary with local norms of community responsibility and governance.

Third, our study focused on reporting engagement as an institutionally enabled form of online bystander intervention. This focus allowed us to theorize how platform social capital is translated into a specific governance-related action. Future research could compare reporting with other bystander responses, such as direct confrontation, supportive messages to targets, counterspeech, or collective mobilization, and examine whether different forms of social capital encourage different intervention strategies. Future studies could also consider situational contingencies, such as content severity, anonymity, visibility of harm, perceived risks of retaliation, and trust in the reporting infrastructure, to clarify when socially embedded users choose reporting rather than alternative responses.

Finally, as platforms increasingly adopt AI-mediated moderation, future work could examine how algorithmic triage, automated feedback, and hybrid human–AI governance systems interact with the social capital dynamics identified in this study. Transparent and responsive moderation systems may strengthen users’ confidence in reporting, whereas opaque or inconsistent outcomes may weaken the perceived value of user participation. Investigating these interactions would further clarify how social and technological infrastructures jointly shape reporting engagement.

## Data Availability

The original contributions presented in the study are included in the article/Supplementary material, further inquiries can be directed to the corresponding author/s.
