# Supplementary material for: Mobilizing the bystanders: How social capital drives user reporting on social media harmful content
Source: Front Psychol. 2026 Jul 13;17:1785107. doi: 10.3389/fpsyg.2026.1785107 (PMC13402366; doi:10.3389/fpsyg.2026.1785107)
Supplement: Supplementary file 1 [file Supplementary_file_1.docx]

**Mobilizing the bystanders: How social capital drives user reporting on social media harmful content**

**Appendix. Constructs and measurement items**

***Reporting Engagement* (Adapted from Schaufeli et al. (2017) and Tsai & Pai (2021))**

**RE1: Reporting harmful content on the platform gives me a sense of energy.**

**RE2: I feel energized when I report harmful content on the platform.**

**RE3: I am motivated to report harmful content on the platform.**

**Social Capital Constructs**

***Structural: Social Interaction Ties* (Adapted from Wang et al.** **(2016))**

**SIT1:** I maintain ongoing contact with users on the platform**.**

**SIT2:** I frequently interact with users on the platform.

**SIT3:** I engage in repeated interactions with users on the platform.

**SIT4:** I tend to interact more with users on the platform.

***Relational: Identification* (Adapted from Chang & Chuang** **(2011))**

**IDF1:** I perceive myself as having established stable relationships with some users on the platform.

**IDF2:** I feel connected to the virtual community on the platform.

**IDF3:** I perceive a sense of unity within the virtual community on this platform.

**IDF4:** I hold generally positive attitudes toward the virtual community on the platform.

***Cognitive: Value Congruence* (Adapted from Tsai & Pai (2021))**

**VC1:** The platform’s rules and reporting features for harmful content are consistent with my personal values.

**VC2:** I agree with the principles reflected in the platform’s reporting rules and features.

**VC3:** The values reflected in the platform’s reporting rules and features are relevant to my use of the platform.

**Mediating psychological constructs**

***Autonomy: Altruism* (Adapted from Mahmood et al. (2019))**

**AT1:** I pay attention to how content on the platform affects other users.

**AT2:** Reporting harmful content allows me to contribute to the platform.

***Relatedness: Sense of Belonging* (Adapted from Zhao et al. (2012))**

**SOB1:** I feel that I belong to this platform.

**SOB2:** I consider myself an involved member of this platform.

***Competence:Reporting Self-Efficacy* (Adapted from Tsai & Pai (2021))**

**RSE1:** My reporting of harmful content can improve the platform experience for other users.

**RSE2:** I am capable of reporting harmful content on this platform.

**Marker variable**

***Learning Cost* (Adapted from Bhattacherjee & Park (2014))**

**LC1:** Becoming familiar with all the features of this platform requires time.

**LC2:** Using this platform requires sustained effort.

**LC3:** Learning to use all functions of this platform is not easy.
